# Trends in the studies of pharmacoresistant epilepsy- a review based on literature analysis (2015–2025)

**DOI:** 10.1186/s42494-026-00249-3

**Published:** 2026-04-02

**Authors:** Menghan Li, Yang Yuan, Junxiu Ye, Xiongfeng Guo, Xin Tian, Cenglin Xu

**Affiliations:** 1 Zhejiang Key Laboratory of Neuropsychopharmacology, Academy of Chinese Medical Sciences, Huzhou Central Hospital, the Fifth School of Clinical Medicine of Zhejiang Chinese Medical University, Zhejiang Chinese Medical University, Hangzhou, Zhejiang 310053 China; 2 Pharmaceutical Engineering College, Jinhua University of Vocational Technology, Jinhua, Zhejiang 321007 China; 3 Department of Neurology, Department of Geriatrics, The First Affiliated Hospital of Chongqing Medical University, Chongqing Key Laboratory of Major Neurological and Mental Disorders, Chongqing Medical University, Chongqing, 400016 China

**Keywords:** Drug-resistant epilepsy, Bibliometrics, Literature analysis, Anti-seizure Medications

## Abstract

Drug-resistant epilepsy (DRE) is a complex neurological disease that accounts for 30%-40% of all epilepsy cases. Its pathogenesis and treatment have always been research hotspots in this field. In recent years, interdisciplinary research on DRE has gradually become a hot topic owing to the research and application of gene sequencing, neuroimaging, new antiepileptic therapies, and artificial intelligence. To reveal the knowledge structure and research trends in this field, this study conducted a bibliometric analysis based on the literature from 2015 to 2025 in the PubMed database, utilizing tools such as R-bibliometrix and CiteSpace to reveal the research trends, core authors, hot topics, and future directions in the DRE field. The analysis showed that from the sorted documents, Professor Bartolomei Fabrice is the most productive author and has published 80 articles on drug-resistant epilepsy. The Capital Medical University of China was the most active institution. The USA, China, and Italy are the countries with the most research contributions, accounting for 25.67%, 15.71%, and 8.09%, respectively. Through keyword analysis and theme evolution, hotspots (such as the ketogenic diet and cannabidiol) and trends (the shift from anti-seizure medications to new therapies) in drug-resistant epilepsy research were explored, revealing dynamic changes in research directions in the field, and that the DRE field is experiencing a paradigm shift from empirical treatment to precision medicine. Robot and artificial intelligence-assisted diagnosis and treatment may become important research directions in the future. This literature analysis study may provide an overview of the research field and references for future research directions in the DRE.

**Supplementary Information:**

The online version contains supplementary material available at 10.1186/s42494-026-00249-3.

## Background

Epilepsy ranks the fourth most common neurological disease globally in prevalence, affecting approximately 70 million people worldwide [1]. Disability-adjusted life years (DALYs) caused by epilepsy account for 1% of the global disease burden [2]. Approximately 30% to 40% of patients with epilepsy (PWEs), do not respond to conventional anti-seizure medications (ASMs), and develop into drug-resistant epilepsy (DRE) [3–6]. DRE not only leads to higher medical expenditures, but also significantly reduces the life quality and increases the risk of mortality, morbidity, and psychosocial dysfunction in PWEs [5, 7]. It has been confirmed that the annual medical expenditure of DRE patients exceeds 26,000 US dollars in the U.S., which is 2 ~ 3 times to that of drug-responsive PWEs [8]. Moreover, patients with DRE usually require more frequent hospitalizations, more complex drug treatment regimens, and possible surgical treatments, which leads to a greater consumption of medical resources. Despite the development of novel ASMs have been proposed, the rate of drug resistance still remain unchanged [9, 10]. For these patients, although neurosurgery and neuromodulation are somehow effective for DRE. However, due to the inapplicability in some DRE patients, as well as the potential side effects, the utilization rate of these therapeutic approaches is still unsatisfied [11].

In recent decades, investigations into the mechanisms and therapeutic approaches for DRE have achieved great advances. The pathogenesis of DRE is complex, with research focusing on several key hypotheses: 1) Neuroinflammation and immune response: a pro-inflammatory microenvironment in the brain activates microglia or astrocytes to release cytokines (e.g., IL-1β, TNF-α) and upregulates HMGB1, which correlates with DRE [12–14]. Astrocyte and microglia dysfunction disrupts the blood–brain barrier (BBB) [15, 16], allowing peripheral immune cells to exacerbate seizures and drug resistance. 2) Transporter hypothesis: Overexpression of Adenosine triphosphate (ATP)-binding cassette (ABC) transporters (e.g., P-glycoprotein) in the BBB effluxes ASMs, reducing their brain concentrations [17, 18]. 3) Genetics and mutations: Somatic mutations (e.g., cortical malformation-related) drive drug-resistant focal epilepsy, while ion channel and synaptic gene mutations are linked to DRE. Genetic susceptibility may also play a role in idiopathic generalized epilepsy [19, 20]. 4) Structural lesions and neuronal remodeling: Hippocampal neuron loss/gliosis in temporal lobe epilepsy (TLE), cortical malformations (e.g., SBH), and abnormal neuronal networks contribute to drug resistance [21, 22]. 5) Neurovascular unit dysfunction: Dysregulation of neurons, glia, and vascular endothelial cells (e.g., ABC transporter overexpression) affects drug distribution [17]. 6) Metabolic and signaling abnormalities: Reduced brain glucose metabolism (e.g., [^18^F]FDG PET hypometabolism) and activated stress kinases (JNK, p38 MAPK) promote neuronal damage [23, 24]. 7) Epigenetic regulation: DNA methylation and histone modifications may alter anti-seizure medication target gene expression (e.g., SCN1A) [25]. 8) Pathological subtypes: Hypothalamic hamartoma (HH) and mild cortical malformation with oligodendrocyte hyperplasia (MOGHE) drive DRE in children. In summary, DRE’s heterogeneity involves neuroinflammation, transporter overactivity, genetic/structural defects, and metabolic abnormalities. Future research should integrate omics and imaging to identify personalized targets, with glial/neurovascular/epigenetic therapies as potential breakthroughs [15, 19, 26, 27].

Management of DRE requires a multidisciplinary approach: 1) Surgery: Resective surgery (e.g., temporal lobectomy) achieves long-term seizure freedom in 60-80% of patients with focal DRE, especially those with structural lesions (e.g., hippocampal sclerosis). Minimally invasive laser interstitial thermal therapy (LiTT) offers precision but remains underutilized globally [28, 29]. 2) Neuroregulation: Vagus nerve stimulation (VNS), deep brain stimulation (DBS), and responsive nerve stimulation (RNS) reduce seizure frequency for ineligible surgical patients. VNS shows efficacy in GAD65 antibody-positive cases [4, 30]. 3) Drug therapy: New ASMs (e.g., levetiracetam, carisbamate, and perampanel) may adjunctively prolong the drug responsive period, but the ultimate drug resistance rates persist. Combination therapy balances efficacy and side effects, although the response is limited in long-term resistant cases [31–33]. 4) Novel therapies: Ketogenic diets reduce seizures in children but require long-term compliance monitoring. Immunotherapies (e.g., targeting HMGB1) and intestinal flora intervention (e.g., FMT) offer new mechanistic insights [30, 34–36].These advances deepen understanding of DRE’s molecular/genetic/structural bases, driving development of targeted treatments.

To deeply understand the current circumstances of DRE research, this study aimed to comprehensively understand the research progress of DRE, reveal research trends and cooperation networks, and provide guidance for clinical and scientific research. This study systematically combs relevant literature from 2015 to 2025 through bibliometric analysis methods, and research hotspots, core countries, institutions, and future directions were identified to reveal knowledge structures and predict future trends.

## Methods

**Fig. 1:**
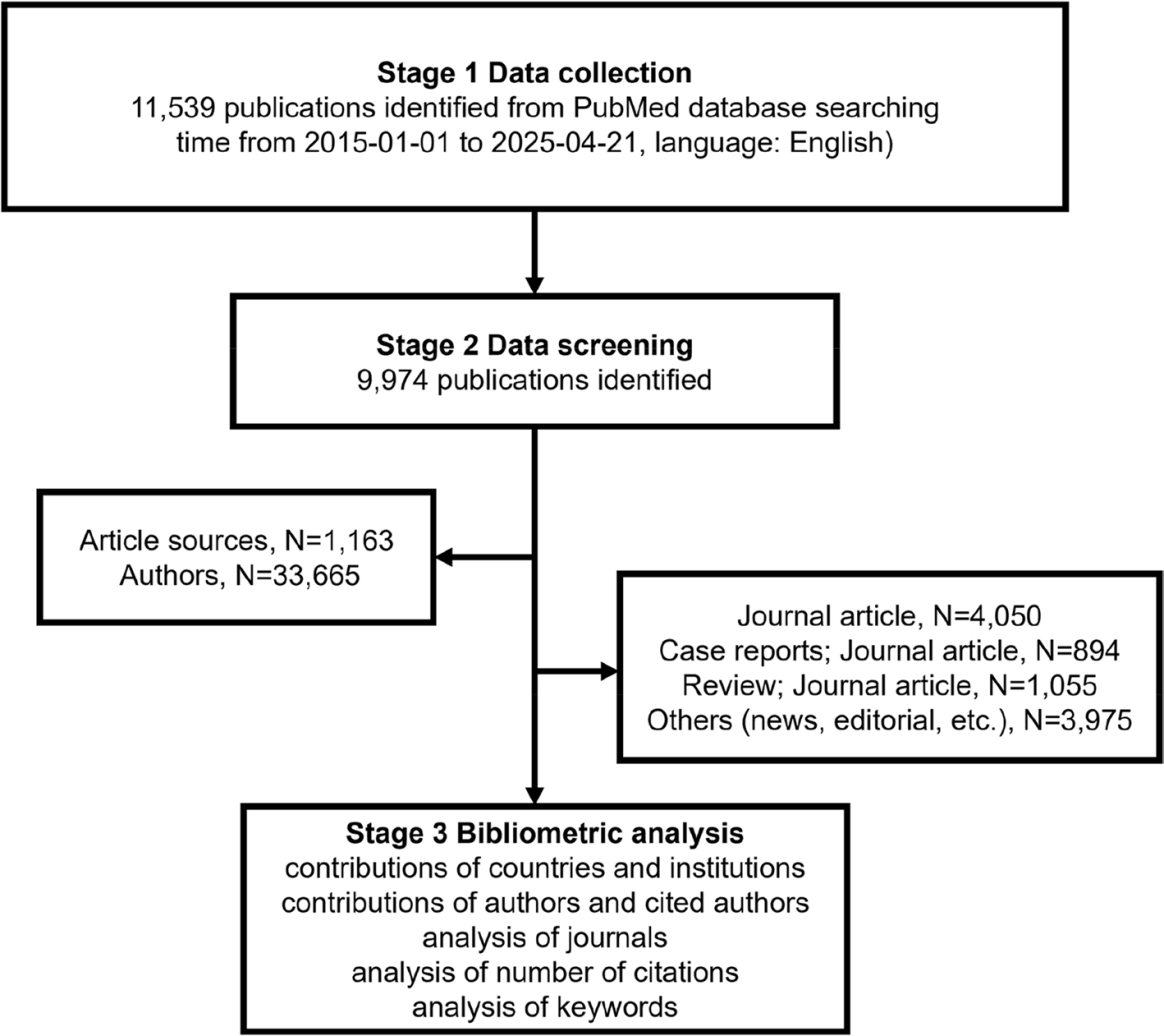
Flowchart of the publication selection

This systematic review retrieved relevant literature from the PubMed database from January 1, 2015, to April 21, 2025 (when manuscript preparation began). The search strategy was (drug resistant epilepsy [MeSH Terms] OR (drug [All Fields] AND resistant [All Fields] AND epilepsy [All Fields]) OR drug resistant epilepsy[All Fields] OR ((pharmacoresistance[All Fields] OR pharmacoresistant[All Fields] OR pharmacoresistent[All Fields]) AND (epilepsie[All Fields] OR epilepsy[MeSH Terms] OR epilepsy [All Fields] OR epilepsies[All Fields] OR epilepsy s [All Fields])) OR (drug resistant epilepsy [MeSH Terms] OR (drug [All Fields] AND resistant [All Fields] AND epilepsy [All Fields]) OR drug resistant epilepsy [All Fields] OR (medication [All Fields] AND resistant [All Fields] AND epilepsy [All Fields]) OR medication resistant epilepsy [All Fields]) OR (drug resistant epilepsy [MeSH Terms] OR (drug [All Fields] AND resistant [All Fields] AND epilepsy [All Fields]) OR drug resistant epilepsy [All Fields] OR (refractory[All Fields] AND epilepsy[All Fields]) OR refractory epilepsy[All Fields]) OR (drug resistant epilepsy[MeSH Terms] OR (drug[All Fields] AND resistant[All Fields] AND epilepsy[All Fields]) OR drug resistant epilepsy[All Fields] OR (intractable[All Fields] AND epilepsy[All Fields]) OR intractable epilepsy[All Fields])) AND (2015:2025[pdat]). Initially, 11,539 articles were identified; after excluding non-English literature, letters, and studies with missing DOI/author information, 9974 articles remained (including journal articles, reviews, meta-analyses, and case reports) from 1163 sources, with 33,665 contributing authors (Fig. 1). Data management used Microsoft Excel (Office 2016). Bibliometric analysis and visualization relied on CiteSpace (Version 5.8.R3, for collaboration/network/co-occurrence/co-citation analysis) and R-bibliometrix (for trending topics and topic maps). Author influence was quantified via H-index, and article impact factors (IF) were calculated using the latest Journal Citation Report [37, 38]. High-frequency word analysis excluded function words (e.g., "the," "of"), domain-general terms (e.g., “epilepsy,” “brain”), and words appearing < 5 times. The unfiltered words can be viewed in Fig.S1.

## Results

### Annual quantitative analysis of enrolled literatures

**Fig. 2:**
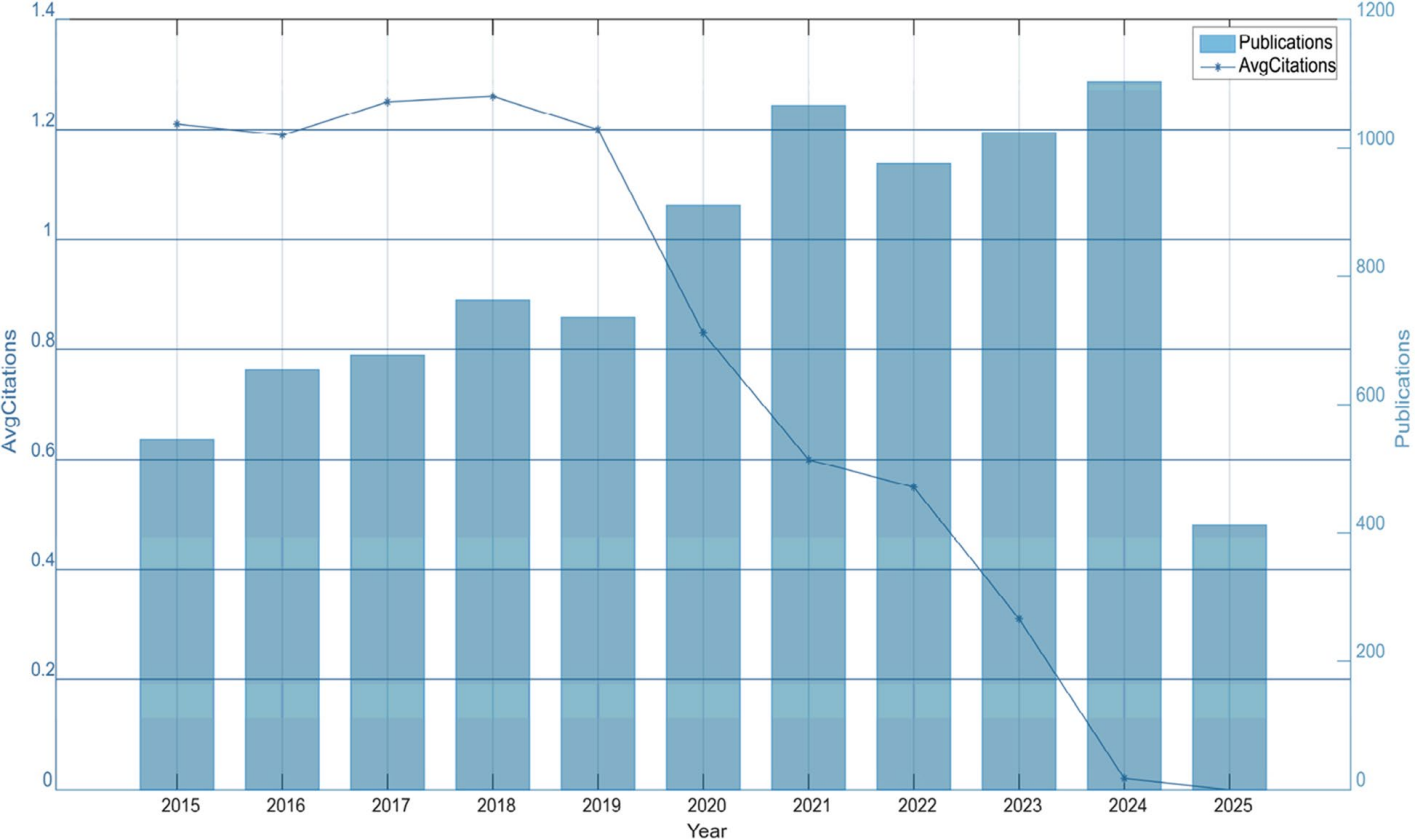
Number of scientific outputs and average number of citations per literature per year

In this bibliometric analysis, we counted the number of publications and citations in different time periods. Figure 2 shows the trends in the number of relevant publications and the average number of citations from 2015 to 2025. The blue bar chart represents the number of publications per year, corresponding to the vertical axis on the right side. From 2015 to 2025, DRE publication numbers showed a steady upward trend, exceeding 400 by April 2025 and remaining at a high level post-2021. Average citations were stable from 2015 to 2018 but declined continuously from 2019 to 2025, nearly dropping to zero in 2024–2025 due to the recency of publications. This reflects dynamic changes in literature output scale and academic influence in the field over time.

### The Countries of origin, institutions, journals and author analysis

**Fig. 3:**
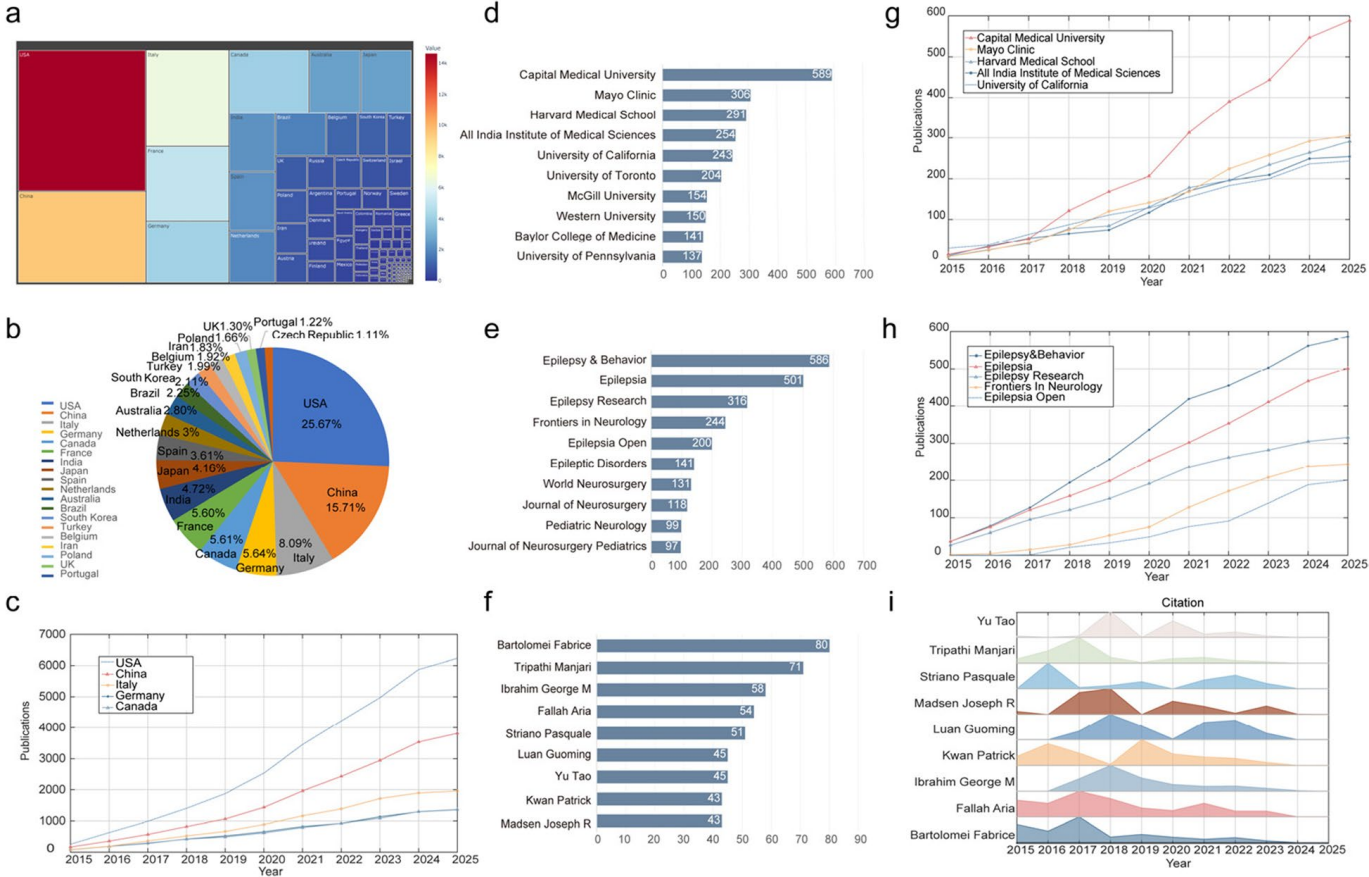
Country journal and author analysis. **a** Treemap of the Distribution of Published Articles by Major Countries Worldwide; **b** proportion of papers published by the top 20 countries; **c**, **e**, **g** number of papers published by the top 5 over time from 2015 to 2025; **d**, **f**, **h** number of articles published in the top 10; **i** citation distribution map of the top 9 authors

DRE research spanned 108 countries/territories and 1379 institutions, the treemap in Fig. 3a shows distribution of the proportion of published articles by major countries worldwide, this chart illustrates the contribution degree of each national unit in the overall structure under the full sample data. The pie chart in Fig. 3b shows the top 20 countries as a percentage of the world's publications. The United States of America (USA) contributed the most publications (25.67%), followed by China (15.71%) and Italy (8.09%), accounting for nearly half of all publications. Figure 3c shows the number of papers published by Canada, China, Germany, Italy, and the USA over time from 2015 to 2025, and the indicator values of all countries show a continuous growth trend. This reflects the increasing input or output of countries in this area over time. In 2015, values were generally low (some in the near single digits), and by 2025, the value of some countries have exceeded 2000 or even reach 6000, showing a significant increasing trend. The USA has contributed particularly most in the publications, followed by China as the second rank. While other countries such as Italy, Germany, and Canada have increased publication numbers, but their growth rate and scale are relatively small.

The publications retrieved were distributed among 1379 institutions, with the top 10 institutions by number of publications, as shown in Fig. 3d, the institution with the highest number of publications was Capital Medical University in China (*n* = 589), followed by the Mayo Clinic in the USA (*n* = 306). Figure 3e shows the institutions that have made the greatest contributions in terms of published journals and their productivity trends over time. The numerical values of all institutional indicators are on an upward trend, reflecting the gradual improvement in the performance of each institution in this field over time. In 2015, the numerical values of most institutions were close to zero or single digits. In 2025, the numerical values of some institutions have reached more than 300 (such as Capital Medical University and the Mayo Clinic). After 2019, the Mayo Clinic exceeded the University of California. Capital Medical University accelerated its growth rate after 2018, and in 2017, it reached the All India Institute Of Medical Sciences. In 2025, the numerical value was 589.

A survey of journals and cited journals showed that three journals on the research progress of DRE had each published more than 300 papers on this topic (Fig. 3f). *Epilepsy & Behavior* led the list with 586 papers, followed by *Epilepsia* (*n* = 501) and *Epilepsia Research* (*n* = 316). Figure 3G shows that the number of publications on DRE in the top 10 journals has increased in the past 10 years. In addition, research in pharmacoepidemiology, clinical pharmacology, and therapeutics has increased.

In the statistics of authors, it was found that 33,665 authors contributed to this research. Figure 3h summarizes the top 9 authors of published papers. Among the top nine authors, all have published at least 40 papers. Bartolomei Fabrice has published 80 papers. Trippathi Manjari, Ibrahim GM, and Fallah Aria published 71, 58 and 54 papers respectively. We also analyzed the total number of citations within 5 years of the top ten authors with the greatest number of papers published on drug-resistant epilepsy (Fig. 3i).

### Cooperation analysis

**Fig. 4:**
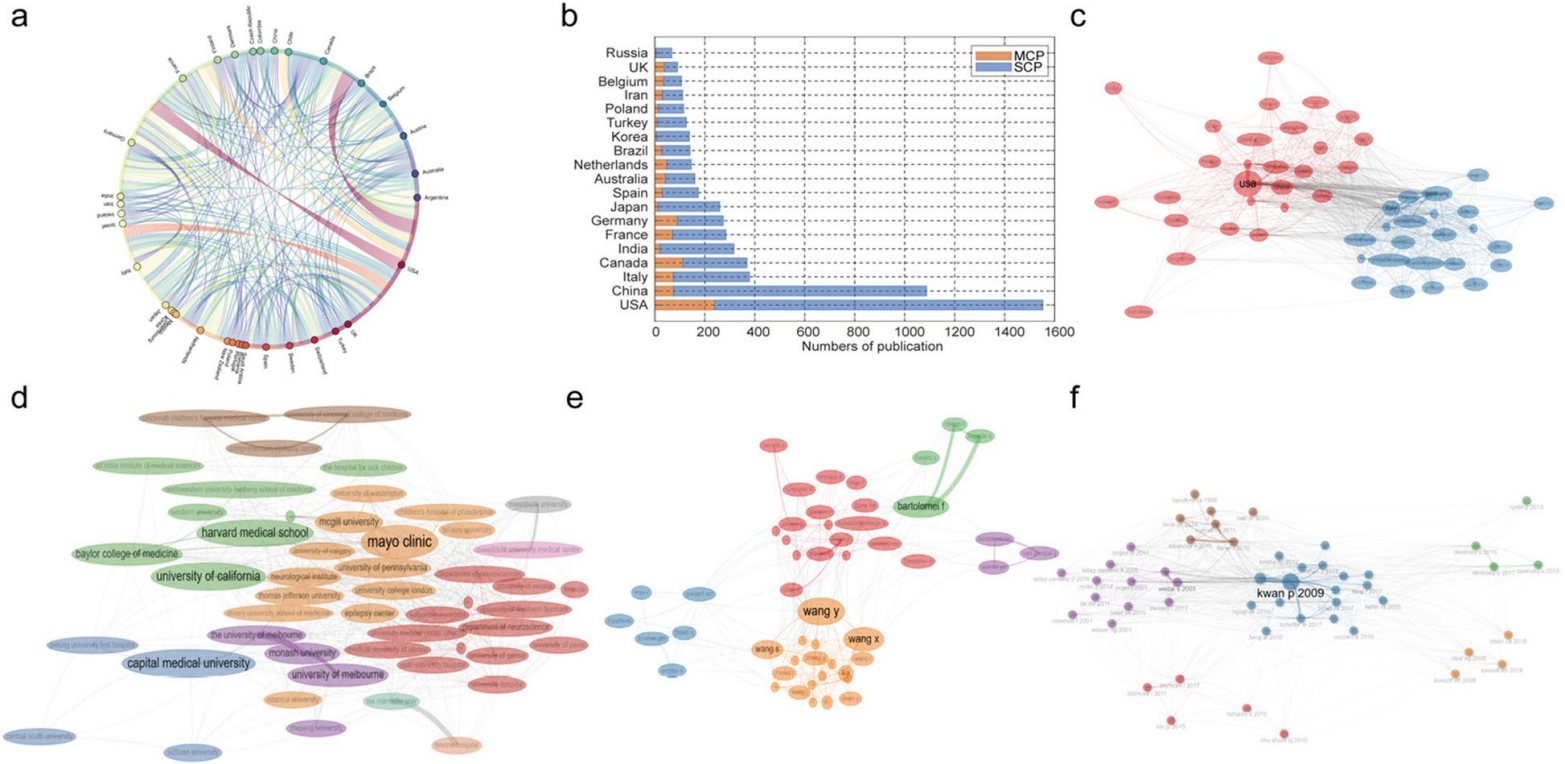
Analysis of Cooperative Relationships. **a** Chord Diagram of Core Interaction Networks Among Global Countries; **b** Cooperation volume of the top 19 countries in terms of the number of papers published. The yellow bars represent the cooperative paper publication volume between countries, and the blue bars represent the paper publication volume of different domestic institutions; **c**-**f** Network relationship diagram, presenting cooperation associations through countries, institutions, and articles

We also considered cooperation between different countries and regions. Figure 4a is the chord diagram of the global inter-country cooperation network after filtering with Top 10% intensity, Warm-colored connecting lines represent high-frequency core interactions, while cool-colored connecting lines represent secondary associations. The bar chart in the Fig. 4b clearly shows that USA, China, and Italy are the countries with the most international cooperation. We then investigated the background of journals and the countries of cited journals. MCP represents the number of papers co-published by authors from different countries, and SCP represents the number of papers co-published by authors from the same country, which illustrates the cooperation between active countries. The USA has the most international cooperation in this field. Although China ranks second in terms of the number of articles, it is not as active as the European countries. The top five country pairs are the USA, Canada, Germany, China, and Italy. The data show that all countries mainly focus on internal cooperation, indicating that international cooperation in drug-resistant epilepsy research is a future trend. We then performed clustering based on the interaction or coupling relationships between documents or authors. Identify which documents or authors in the document dataset have coupling relationships, such as mutual citation, co-cooperation, or common attention, and classify them into different groups or categories. The collaboration networks (Fig. 4c-f) are mapped to different clusters, and nodes are divided by colors such as red, blue, purple, green, and orange. Each color in the network represents a cluster or group of collaborating. In Fig. 4c, the map can be clearly divided into 3 types of collaborative clusters. The core nodes in the red cluster are the USA and China, covering the Americas (such as Brazil, Mexico, and Cuba), East Asia (such as Japan, Korea, and Singapore), Southeast Asia (such as Malaysia, Indonesia, and Vietnam), South Asia (such as India and Pakistan), and some Middle Eastern countries (such as Saudi Arabia), with the USA as the “super hub” and China as the “regional core”, relying on the shipping and economic connections of the Pacific and Indian Oceans, a wide cross-continental cooperation network is formed, reflecting the cooperation logic of “geoeconomic drive and complementary disciplinary needs”. The core nodes in the blue cluster are the USA, Germany, and France. Centered on Western Europe, it radiates to Eastern Europe (such as Hungary, Bulgaria, Croatia, etc.), Northern Europe (such as Sweden, Finland, etc.), and Southern Europe (such as Portugal, Greece, etc.), rely on the EU research framework to form a “Regional Integration Collaboration Network”, with close connections among core nodes. Cross-cluster transition zone includes some countries that connect the pink and blue clusters, assume the role of the “bridge for cross-regional collaboration”, and promote scientific research interactions between the Pan-American-Asia-Pacific cluster and the European cluster. Figure 4d is Collaboration Network Map of Scientific Research Institutions in the Medical Field, presenting the collaborative connections among scientific research institutions in global epilepsy (or neuroscience-related) research.

Mayo Clinic, Harvard Medical School serving as the “Global Collaboration Engine”, connecting cross—cluster institutions and leading international multi—center research (such as large—scale epilepsy clinical cohorts and research on neural regulation technologies). University of California, Baylor College of Medicine, etc., serve as “Regional Collaboration Centers”, connecting the core hubs with local institutions (for example, the University of California links up with the scientific research network in the western USA). Characteristic institutions of various countries (such as Capital Medical University in China and Monash University in Australia) assume the role of “localized collaboration” and promote regional research (such as Asian epilepsy epidemiology and European neuroimaging technology). Green Cluster (centered around Harvard Medical School): Focus on basic neuroscience (such as the pathogenesis of epilepsy and neural circuit research), rely on Harvard’s scientific research resources (such as the Broad Institute and the Center for Brain Science), and collaborate with basic research institutions worldwide. Orange Cluster (centered around Mayo Clinic): Emphasize clinical translational research (such as epilepsy surgical treatment and precision drug trials), leverage Mayo’s clinical resources (multispecialty collaboration, patient cohorts), and promote the “research-clinical” closed loop. In Fig. 4e, the orange cluster, Wang Y, Wang X closely cooperate with Wang S, Zhang Y, etc.; in the red cluster, Verrotti A is associated with Aronica E, Specchio N, etc., and multiple parties collaborate with each other; the green cluster takes Bartolomei F as the core and cooperates with Rheims S, Carron T, etc.; in the purple cluster, Lundstrom BN connects with Wonnel GE, Van Gompel JJ; in the blue cluster, Arya R, Gaillard WD, Tripathi M, Ibrahim GM, etc. collaborate with each other. Each cluster forms a scientific research cooperation network through the connections among its members. The network we constructed based on the co-citation relationship among the documents in the dataset identifies the documents that are frequently cited together in the document dataset to create a co-cited document network, as shown in Fig. 4f. Among the 50 co-cited references obtained, the article “Kwan P., 2009, The Lancet Neurology” is an important article with the highest citation frequency and relatively high influence in this field. The analysis results show a strong co-citation relationship between this article and “kwan p., 2000”, “scheffer ie., 2017”, “fisher rs., 2017” and “chenz., 2017”.

### Keyword analysis

**Fig. 5:**
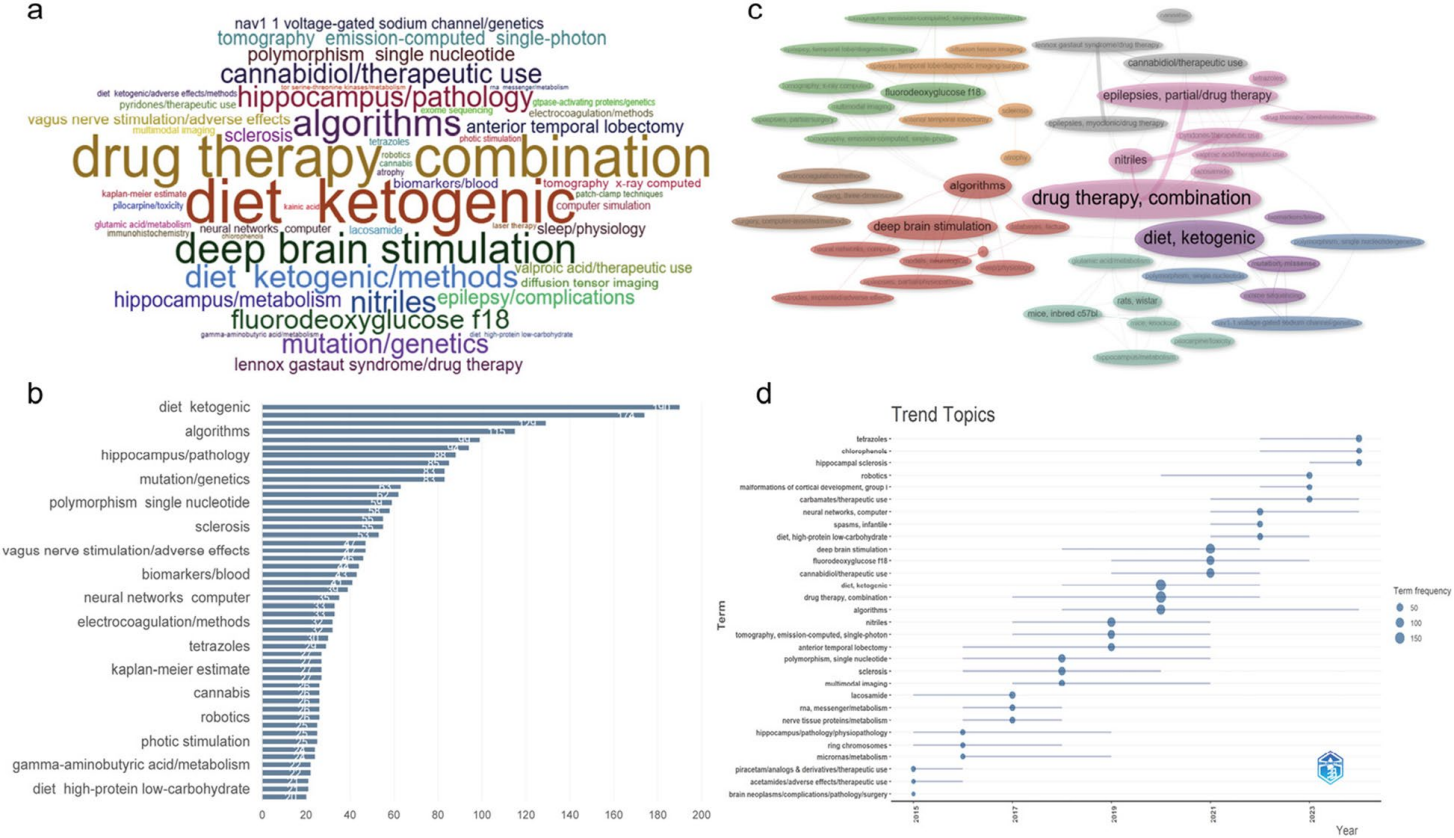
Keyword analysis. **a** Word cloud after removing duplicate words; **b** Sorting of the Occurrence Times of High-Frequency Vocabulary; **c** The correlation between high-frequency words; **d** Trend graph of the emergence of different topics from 2015 to 2025. The lines represent time across years, and the size of the dots represents the frequency of the topic's appearance

To facilitate drug-resistant epilepsy (DRE) research trend analysis, we excluded general terms (e.g., “human,” “cell,” “epilepsy”) from 13,598 keywords (pre-exclusion analysis in Fig.S1-S2). Figure 5a’s R-Biblioshiny-generated word cloud (font size = frequency) highlights top terms: “diet ketogenic” (190 occurrences), “drug therapy, combination,” “deep brain stimulation,” “cannabidiol,” “nitriles,” “algorithms,” and “hippocampus,” with emerging terms like “tetrazoles,” “chlorophenols,” “robotics” and “hippocampal sclerosis.” In Fig. 5b, we have sorted the top 50 keywords, the relationship between different keywords in drug-resistant epilepsy research is visually presented. In Fig. 5c visualize keyword relationships and clusters (color-coded): pink (drug therapy, e.g., cannabidiol), purple (ketogenic diet), green (basic research/animal models), orange (pathology/surgery), red (technology/algorithms), gray (specific substances). Node size indicates frequency (“drug therapy, combination” as core), and line thickness reflects co-occurrence. Figure 5d covers the period from 2015 to 2025 and is used to present the research popularity of each subject term in different years (through Term frequency, that is, the term frequency, and the node size corresponds to frequencies of 50, 100, and 150), reflecting the trend changes of research topics in epilepsy and related fields over time. The “diet ketogenic” rose post-2018, integrating with drug therapy for pediatric DRE; “drug therapy, combination” linked to cannabidiol post-2022; DBS gained traction 2020–2022, merging with AI/robotics; multimodal imaging (2017–2020) aided focus localization; cannabidiol (2019–2021) expanded to molecular mechanisms; genetics research advanced to polygenic susceptibility; robotics emerged in 2022, reflecting “AI and Neurology” innovation.

**Fig. 6:**
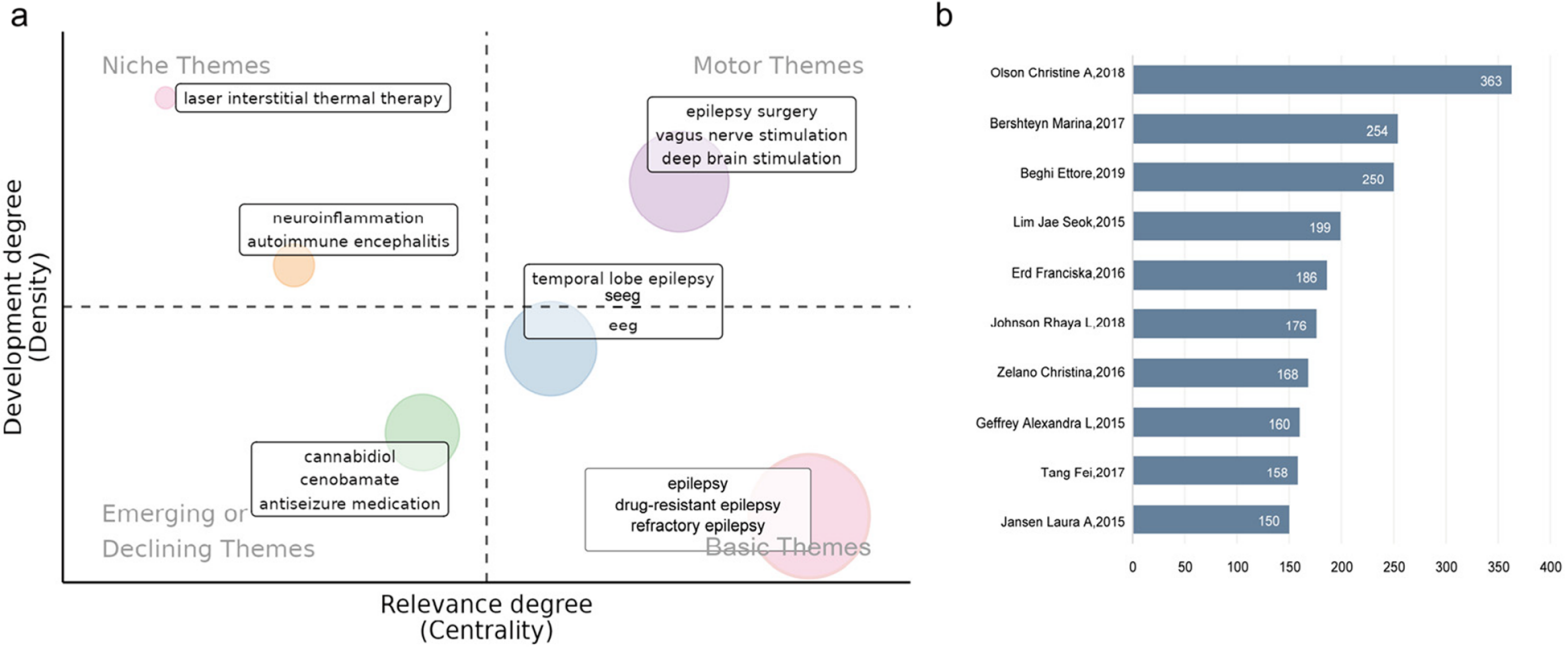
**a** Topological regions of keywords plus, characterized by two parameters: development degree (density) and relevance (centrality); **b** Sorting the number of citations from low to high

Then we divide keywords plus into four topological regions (Fig. 6a) and characterize them with two parameters: density and centrality are depicted by two parameters of “development degree (density)” and “relevance (centrality)”. Different themes are represented by circles of different colors. The size of the circles represents indicators such as the number and popularity of research related to the theme, to understand the relative status of each theme. The horizontal axis represents the degree of relevance (centrality). The degree of relevance gradually increased from left to right, reflecting the closeness of the connection between the theme and other research content. The vertical axis represents the degree of development (density). The degree of development increases gradually from bottom to top, reflecting the maturity or degree of attention of the theme in the research. The upper right quadrant (Motor Themes) includes “epilepsy surgery”, “vagus nerve stimulation”, and “deep brain stimulation”. These themes have a high degree of correlation and development, and are the core driving themes in the field of epilepsy research, representing important treatment methods that are mature and closely related to other researches. The lower—right quadrant (Basic Themes) covers “epilepsy”, “drug—resistant epilepsy”, and “refractory epilepsy”. The degree of correlation is high, but the development level is relatively basic. It is the fundamental and core theme of epilepsy research and the cornerstone for the conduct of other researches. The lower left quadrant (Emerging or Declining Themes) includes “cannabidiol”, “cenobamate”, and “antiseizure medication”. Both the degree of association and development are relatively low, representing emerging research directions. The upper left quadrant (Niche Themes) includes “laser interstitial thermal therapy”, as well as “neuroinflammation” and “autoimmune encephalitis”. These themes have a high degree of development but low correlation. They are research topics with strong professionalism and are relatively niche, with in-depth development in specific fields.

Finally, we sorted the cited documents from lowest to highest and presented them in Fig. 6b. It is used to present the citation frequency of documents published by different scholars in the corresponding years, reflecting their academic influence. Among them, the document published by Olson Christine A in 2018 has the highest number of citations, while the documents published by Jansen Laura A and others in 2015 and other years have relatively lower citation numbers.

## Discussion

### Global research distribution

DRE research output is highly unbalanced: 108 countries participated, but the U.S., China, and Italy dominated. The U.S. benefits from strong biomedical infrastructure (e.g., NIH funding) and top institutions (Mayo Clinic, University of California) [39]; China’s rapid growth stems from national policies (e.g., Brain Science Research Program) and institutional advancement (e.g., the Capital Medical University) [40]. All countries showed growth (2015–2025), driven by clinical needs (30–40% DRE rate), technological innovation (neuromodulation), and policy support (China’s “Brain Project,” EU Horizon). However, a “Matthew Effect” persists: low-income countries (e.g., Africa) are underrepresented, requiring equitable participation via international cooperation and funding [41]. However, a “Matthew Effect” persists in this field of study. Some resource-poor countries (such as those in Africa) are almost excluded from the research systems. In the future, more equitable research participation must be achieved through international cooperation and financial assistance.

### Institutional contributions and scientific research collaboration models

Among the 1,379 research institutions globally, Capital Medical University (589 papers) and Mayo Clinic (306 papers) topped the list (Fig. 3d), indicating a high concentration of academic resources. Considering the temporal evolution (Fig. 3g), US institutions were dominant from 2015 to 2019; however, after 2018, Chinese institutions (such as Capital Medical University) accelerated their growth rate and even surpassed some European and American institutions. This phenomenon suggests that China's competitiveness in neuroscience is increasing, possibly due to national strategic support and the return of scientific research talent. In terms of international cooperation, the USA plays a central role (Fig. 4a–b), collaborating closely with Europe (Germany and Italy) and Asia (China). However, although China ranks second in the number of papers, its international cooperation activity is relatively low, indicating that its research is mainly domestic. It is worth noting that most cooperation focuses on clinical trials, cohort studies, and randomized controlled trials (Fig. 4c), because these studies usually require multi-center data. Nevertheless, “same-country cooperation” (SCP) still dominates, indicating that international collaboration has not yet become mainstream. Possible reasons include: data-sharing barriers, such as inconsistent patient privacy regulations in different countries that hinder cross-border research; resource competition, where institutions and researchers may be more inclined to prioritize the publication of domestic results; and lack of standardization, where the diagnosis and treatment of DRE have not been fully unified, increasing the difficulty of international cooperation. In the future, a global epilepsy research alliance should be established to promote Open Science and standardized data-sharing agreements to facilitate cross-border collaboration.

### Analysis of the influence of journals and authors

Leading journals (*Epilepsy & Behavior*, *Epilepsia*, *Epilepsy Research*) show growing DRE publications, indicating active academic exchange. Emerging journals (e.g., *Pharmacoepidemiology and Clinical Pharmacology & Therapeutics*) reflect new focuses on pharmacoeconomics and treatment optimization. Top authors (Bartolomei Fabrice, Trippathi Manjari) drive clinical research clusters, but collaboration remains within institutions/countries. Highly cited papers focus on surgery, neuromodulation, and drug efficacy that validating clinical research’s dominant role.

### Research on the evolution of research topics and cutting-edge trends

DRE research has undergone three key transitions (2015–2025): 1) Treatment strategies: From traditional drug combinations/ketogenic diets to advanced neuromodulation (DBS/VNS optimization, LiTT) and intelligent integration (robotics, AI); 2) Disciplinary model: From single neurology to “AI + neuroscience” (e.g., robot-assisted surgery, AI dynamic regulation); 3) Mechanism understanding: From macroscopic symptom control to multi-omics analysis (neuroinflammation, single nucleotide polymorphisms for precision intervention).Current cutting-edge directions include: 1) AI-brain-machine interfaces (seizure prediction, adaptive neuromodulation); 2) Spatial omics and immune microenvironment research (unlocking resistance mechanisms); 3) Plant-derived drugs (cannabidiol and traditional ASMs), metabolic interventions, and neural circuit regulation; 4) Surgical robots with 7 T-MRI (precise ablation); 5) Single-cell omics (drug resistance analysis); 6) Wearable devices (real-world evidence). Key challenges: clinical translation gaps (algorithm generalization, lack of elderly/pregnant population data), mechanism-application disconnection (neuroinflammation research lagging in therapy), and global resource imbalance (low-income regions’ 5 + year technology gap). Future progress requires a “multi-omics mechanisms—intelligent spatiotemporal intervention—dynamic monitoring—ethical safety” collaborative network to shift DRE care from empirical to precise, closed-loop management.

### Limitations of the current research in DRE

Recent advances in DRE management highlight transformative progress alongside persistent translational gaps. At the diagnostic level, the definition by the ILAE lacks unified clinical implementation standards [42]. Although whole-exome sequencing (WES) has significant value for the early diagnosis of infants and young children, its widespread application is limited by costs and the popularity of the technology [43, 44]. At the treatment level, more than 20 new antiseizure medications approved over the past 20 years are ineffective for about 1/3 of PRE patients, and the problem of drug resistance has not been fundamentally resolved [45]. Precision medical technologies such as gene editing and viral vector delivery are still in the early stages of clinical trials, and their safety and efficacy remain to be verified [46, 47]. The efficacy of neural intervention methods like VNS and DBS, as well as epilepsy surgeries in complex locations, is constrained by individual response differences and the accuracy of epileptic focus localization [48, 49]. Comorbidities (such as depression and intellectual disability) and individual differences mediated by genetic background further increase the difficulty of personalized treatment [50]. Additionally, the differences between animal models and human physiology and pathology, along with ethical and policy constraints, constitute the “last mile” obstacle in translational research [51].

## Conclusions

This study presents a global research landscape in DRE from 2015 to 2025 through bibliometric analysis, examining publications, citations, country/region contributions, and cooperation networks. Through keyword analysis, it identifies research hotspots like ketogenic diet and cannabidiol, and trends shifting from anti-seizure medications to new therapies, showing the field's evolution from empirical treatment to precision medicine. It highlights the USA, China, and Italy's leading positions while noting China’s limited international cooperation, providing direction for future collaboration. The results offer reference for academia and basis for clinical practice and policy. The research focus evolved from drug combinations and ketogenic diet (2015–2018) to neuromodulation technology (2019–2022), and then to intelligent closed-loop diagnosis systems (2023–2025), progressing from symptom control to neural circuit remodeling. Current research priorities include AI-brain-machine interface for seizure prediction, surgical robots with 7 T-MRI for precise ablation, drug-resistance analysis through single-cell omics, integration of antibody drugs and neuromodulation, and real-world evidence systems using wearable devices. Future challenges include algorithm generalization, evidence gaps for special populations, global resource imbalance, and ethical issues with brain-machine interfaces. The core direction aims to build a network of “multi-omics mechanisms—intelligent intervention—monitoring—ethical safety” to advance DRE treatment through interdisciplinary integration.

However, this review was exclusively based on the PubMed database, potentially overlooking literature from other sources, such as the Web of Science Core Collection. The inclusion of only English-language literature may result in the omission of significant research from non-English-speaking countries. Furthermore, the inclusion of various types of case reports may obscure research focal points, and errors in author analysis may arise due to authors sharing identical names. In future research, more efforts can be made to supplement the above shortcomings.

## Supplementary Information

Supplementary Material 1.

## Data Availability

Not applicable.
