# Supplementary material for: Trends in the studies of pharmacoresistant epilepsy- a review based on literature analysis (2015–2025)
Source: Acta Epileptol. 2026 Apr 2;8:10. doi: 10.1186/s42494-026-00249-3 (PMC13045044; doi:10.1186/s42494-026-00249-3)
Supplement: Supplementary file 1 — Supplementary Material 1. [file 42494_2026_249_MOESM1_ESM.docx]

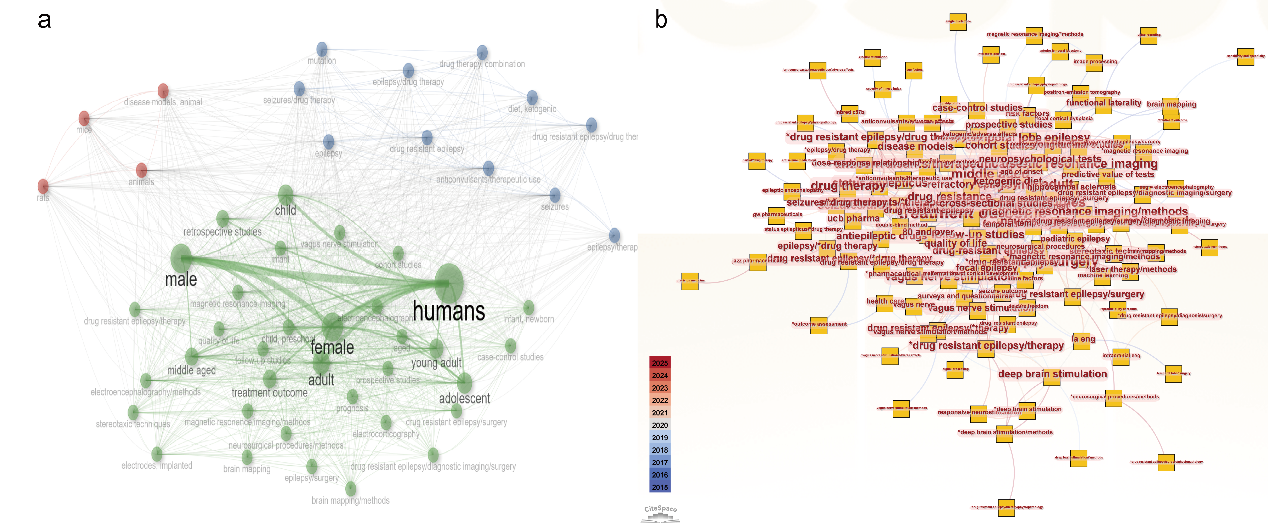


**F I G U R E S 1** Keyword analysis. a) Word cloud distribution before removing interfering words; b) Analysis distribution of professional terms in CiteSpace.


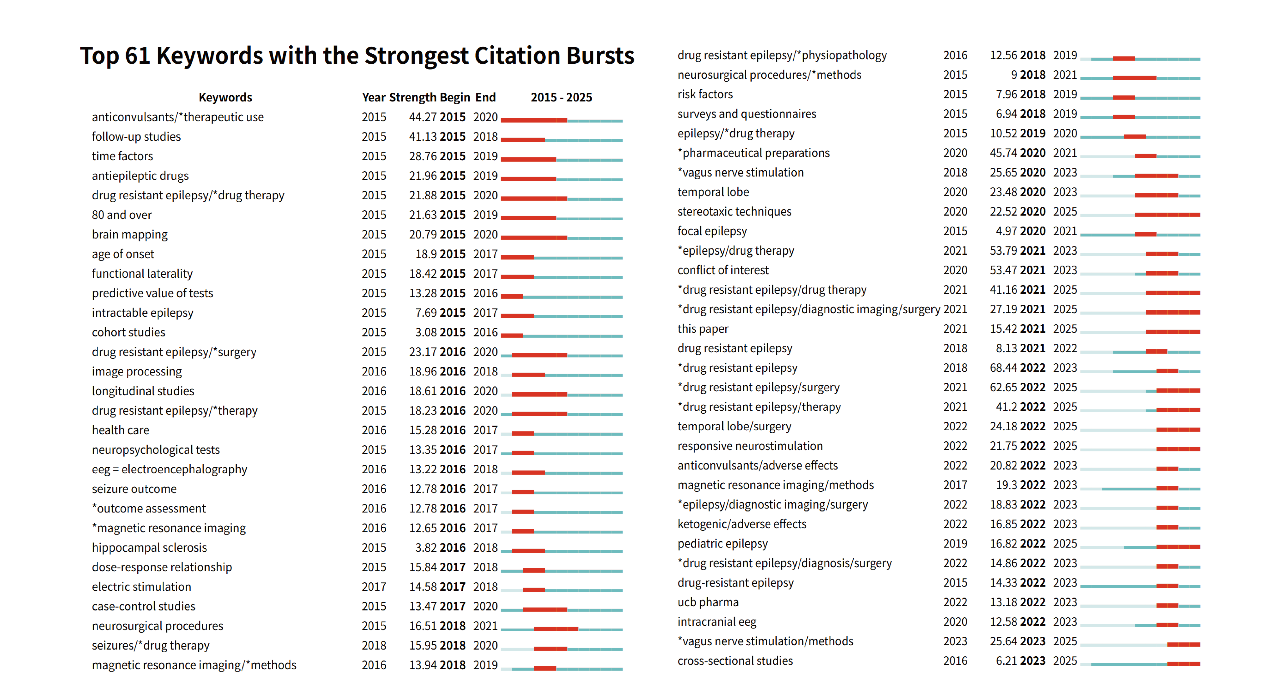


**F I G U R E S 2** High-frequency vocabulary analyzed by CiteSpace and their respective prominent duration and intensity.
